# Lower Limb Posture Affects the Mechanism of Injury in Under-Body Blast

**DOI:** 10.1007/s10439-018-02138-4

**Published:** 2018-10-01

**Authors:** Grigoris Grigoriadis, Diagarajen Carpanen, Claire E. Webster, Arul Ramasamy, Nicolas Newell, Spyros D. Masouros

**Affiliations:** Department of Bioengineering, Imperial College London, South Kensington Campus, London, SW7 2AZ UK

**Keywords:** Blast injury, Trauma biomechanics, Post mortem human surrogates, Anthropometric testing devices, Underbody blast, Lower limb biomechanics, Foot and ankle, Lower limb posture

## Abstract

Over 80% of wounded Service Members sustain at least one extremity injury. The ‘deck-slap’ foot, a product of the vehicle’s floor rising rapidly when attacked by a mine to injure the limb, has been a signature injury in recent conflicts. Given the frequency and severity of these combat-related extremity injuries, they require the greatest utilisation of resources for treatment, and have caused the greatest number of disabled soldiers during recent conflicts. Most research efforts focus on occupants seated with both tibia-to-femur and tibia-to-foot angles set at 90°; it is unknown whether results obtained from these tests are applicable when alternative seated postures are adopted. To investigate this, lower limbs from anthropometric testing devices (ATDs) and post mortem human subjects (PMHSs) were loaded in three different seated postures using an under-body blast injury simulator. Using metrics that are commonly used for assessing injury, such as the axial force and the revised tibia index, the lower limb of ATDs were found to be insensitive to posture variations while the injuries sustained by the PMHS lower limbs differed in type and severity between postures. This suggests that the mechanism of injury depends on the posture and that this cannot be captured by the current injury criteria. Therefore, great care should be taken when interpreting and extrapolating results, especially in vehicle qualification tests, when postures other than the 90°–90° are of interest.

## Introduction

Improvised explosive devices (IEDs) have been identified as the most prevalent cause of injury in modern warfare.^10^,^32^–^34^ Mounted victims of IED attacks sustain severe, difficult-to-treat injuries that result in high rates of amputation.^35^ These injuries mainly include intra-articular calcaneal and tibial fractures and occur as the floor of the vehicle deforms rapidly, transferring axial loading to the lower limb of the occupants.^6^,^9^,^36^

Various platforms have been developed previously to replicate underbody blast (UBB)—that is, the attack of the underside of a vehicle by explosives—in the laboratory and used to test surrogate lower limbs such as post mortem human surrogates (PMHSs) and anthropometric test devices (ATDs).^1^,^7^,^12^,^13^,^15^,^19^,^21^,^23^,^31^,^46^,^47^ To assess injury, the results from these tests have been analysed to develop injury risk functions (IRFs), which relate the probability of injury with the axial force transmitted to the lower limb.^2^,^3^,^23^,^24^,^43^,^45^ Although parameters such as age, gender, and bone mineral density,^12^,^45^ as well as loading rates and location of measurements have been taken into account when developing IRFs,^2^ the effect of the posture of the occupant at the time of the attack on the injurious outcome has not.

**Figure 1:**
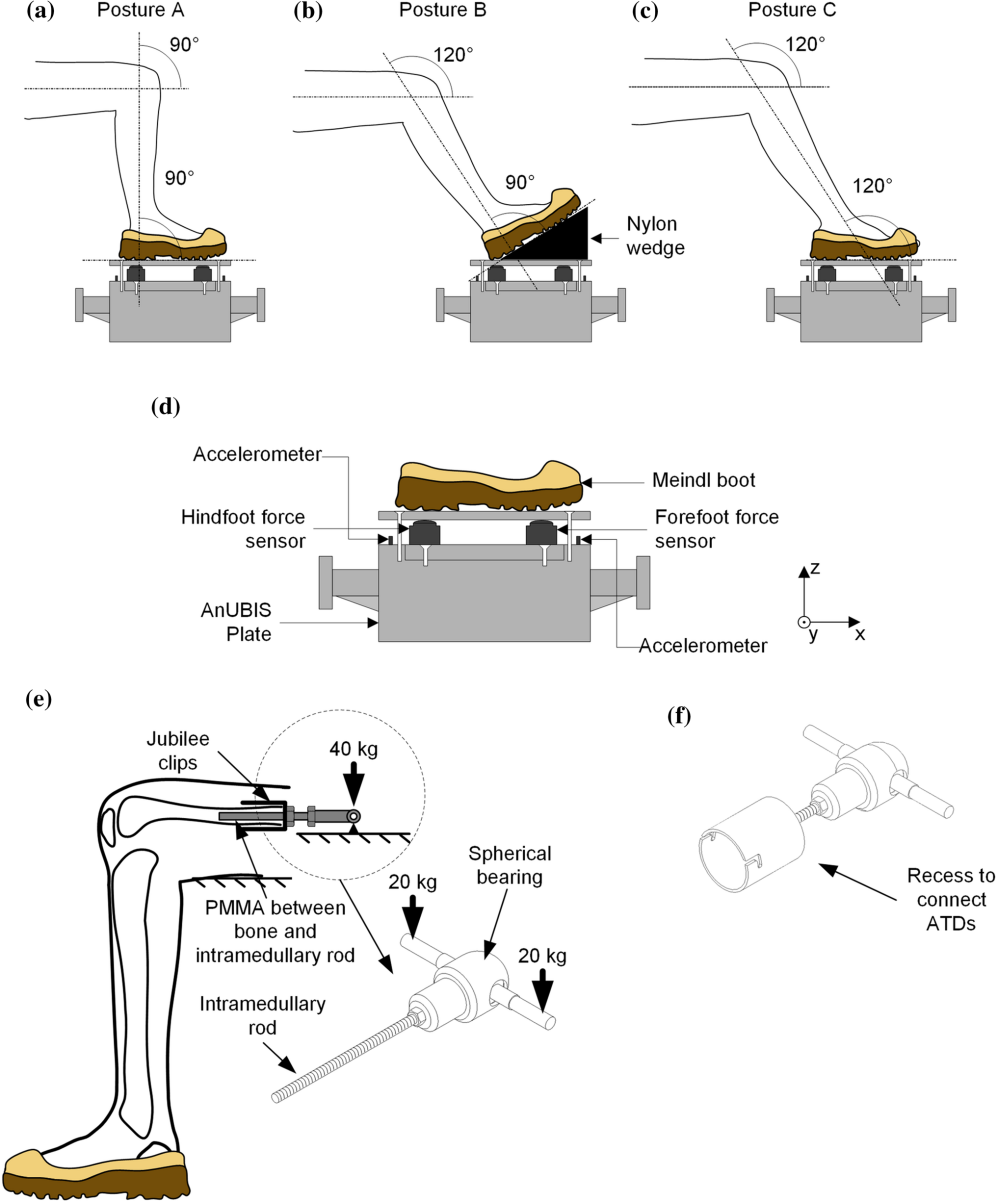
The three seated postures examined in this study; (a) 90°–90°, (b) 120°–90°, (c) 120°–120°. (d) Diagram of the experimental setup used in this study. Schematics showing the method to mount the (e) PMHSs and (f) ATDs on AnUBIS, which was modified from Masouros *et al*.^21^

The boundary conditions applied in most previous studies consider the 90°–90° posture where both the angle of the foot to the tibia and the tibia to the femur, defined in this study as the knee and ankle angles, respectively, are set at 90° (Fig. 1a). Yoganandan *et al*.^45^ impacted dorsi-flexed specimens with a pendulum but combined the results with outcomes from impacting specimens set at the 90°–90° posture to derive the IRF that is used in the NATO standard for vehicle qualification.^28^ A pendulum apparatus was also used by Klop *et al*.^16^ to impact PMHS lower limbs with the ankle joint set at various dorsiflexion angles with results showing that dorsiflexion of the ankle joint decreases the probability of injury. However, this experimental setup refers to the posture of the driver of a vehicle in a road traffic accident, with the foot mounted on a brake pedal and the force transmitted in the direction of a frontal vehicle collision, which is not a representative loading environment in UBB.

Newell *et al*.^25^ performed tests on lower limbs of ATDs set in both seated and standing postures demonstrating that posture alters the injurious outcome drastically, with the standing posture resulting in more severe injuries. This was in agreement with the severity of injuries reported from testing PMHSs in seated and standing postures.^20^ Pandelani *et al*.^30^ used a spring mechanism to test two different lower limb ATDs, the Hybrid III (H-III) lower limb and the Military Lower Extremity (MiL-Lx), in various seated postures. While axial tibial force measured by both ATDs was greater when they were set at the 90°–90° posture, the Revised Tibia Index (RTI)^17^ was found to be greater when the knee angle was set at an angle greater than 90°. However, no PMHS studies were conducted to further investigate this finding or inspect any other alternative seated postures that are likely to be adopted by occupants in military vehicles as suggested in previous reports.^27^,^38^ Therefore, the aim of this study was to use lower limb surrogates, including both PMHSs and ATDs, to investigate whether the posture of the lower limb of the occupant affects the injurious outcome in a UBB incident.

## Materials and Methods

The Antivehicular Under-Belly Blast Injury Simulator (AnUBIS) previously developed at Imperial College London^21^ was used to perform in total 28 tests; 9 with the H-III, 9 with the MiL-Lx, and 10 with PMHSs. The three different postures that were investigated in this study are shown in Figs. 1a–1c.

The top plate of AnUBIS was instrumented with two single-axis force sensors (model 200C20, PCB Piezotronics Ltd, Hitchin, UK) that measured the force transmitted to the hind-, *F*_H_, and the fore-foot, *F*_F_, of the surrogate lower limb in the direction of the impact, and with two uniaxial accelerometers (model 350D02, PCB Piezotronics Ltd, Hitchin, UK) as shown in Fig. 1d. The plantar force was calculated as the sum of the hind-foot and fore-foot forces. On top of the force sensors, a single, 15 mm thick mild steel foot plate was mounted with 4 bolts and was measured to be parallel to the top plate of AnUBIS with an inclinometer (model SlopeView TLL-90E, MIB Instruments Co, Hong Kong SAR). The foot plate was behaving rigidly, as assessed by analysing the high-speed video footage; deflection was not observed in the fastest tests (the resolution was 0.3 mm). This was expected as the force was concentrated under the heel and the metatarsal heads regions. Apart from these alterations, the setup of the rig was the same as described by Masouros *et al*.^21^; a 16 kg plate was mounted on a pressure vessel with a tie rod secured through a cross-pin. As air was fed into the vessel, pressure built up to the point that the cross-pin failed due to double shear. The AnUBIS plate was then free to travel upwards for 50 mm before being decelerated by 4 tapered side braking arms. Depending on the material and diameter of the cross-pin different threat characteristics can be simulated; in this study, brass and mild steel pins of diameters of 9, 11, 12.7 mm were used for testing ATDs and PMHSs, respectively.

The total mass of the accelerated plate under the surrogates was 18 kg. Two high speed cameras (Phantom V12.1 and V611, 8000 fps, Vision Research, Bedford, UK) were used to capture all tests focusing on the medial and lateral sides of the ankle-joint complex of all surrogates. All data were recorded at a frequency of 25 kHz using a PXIe data acquisition system (model 1082, National Instruments, Austin, TX, USA) and a custom-written LabVIEW code (v2012, National Instruments, Austin, TX, USA). The signals from the accelerometers were filtered using a CFC 1000 filter, as suggested by standards for vehicular impact testing,^40^ and integrated once and twice to calculate the velocity and displacement profiles of the plate for all tests, respectively. The peak acceleration and velocity values as well as the time to peak velocity were averaged over tests on each surrogate; H-III, MiL-Lx, and PMHS.

The thigh of all surrogate limbs was attached through a spherical joint, replicating the motion of the hip joint, to a rod supporting a weight of 40 kg, to represent half the body weight of the occupant, as described by Masouros *et al*.^21^ The femoral heads of the PMHSs were removed and an intramedullary rod was inserted in the femoral bone and secured using polymethyl methacrylate (PMMA) and two jubilee clips. The proximal end of the intramedullary rod was threaded to the spherical joint connected to the weights that were resting on AnUBIS (Fig. 1e). The ATDs were connected similarly using the two protruding cylindrical pins at the proximal end of their thighs (Fig. 1f).

The knee and ankle angles for all surrogate limbs were measured using a digital inclinometer (model SlopeView TLL-90E, MIB Instruments Co, Hong Kong SAR). The measurements were taken between the long axis of the tibia, the long axis of the femur, and the surface on which the foot was resting. For the PMHSs, the long axis of the tibia was determined as parallel to the axis between the medial malleolus and the medial tibial epicondyle, while for the ATDs it was defined as the axis between the ankle clevis with the knee clevis at the sagittal plane. The long axis of the femur was defined as the axis of the intramedullary rod for the PMHSs, and the long axis of the cylindrical mount at the proximal end of the thigh for the ATDs. The femoral long axis was always set to be parallel to the surface on which the foot was resting. To achieve posture 120°–90° in tests with either ATDs or PMHSs a nylon wedge of an angle of 30° and mass of 1.2 kg was manufactured and secured on the AnUBIS plate below the foot. A UK size 10 Meindl Desert Fox Combat boot (Lukas Meindl GmbH & Co., Kirchanschöring, Germany) was fitted to each of the ATDs before every test. The same boot was also used for the PMHSs but the sizes varied from 6 to 10, to ensure good fit with the specimen. For the PMHS tests, side openings were made to the lateral and medial sides of the boot to allow the high speed camera to record both sides of the calcaneal bone. The rest of the boot was left intact and it was tightened using the shoe laces provided. The measured variables from all tests were averaged per posture, compared using ANOVA, and analysed *post hoc* using a Games-Howell test with the significance level set at *p* = 0.05 (SPSS Statistics, Version 22.0, IBM Corp., Armonk, NY).

The lower limb of the 50th percentile H-III ATD was instrumented with both upper and lower tibia load cells by the manufacturer to measure axial force, *F*_Z_, shear force in the anterior–posterior direction, *F*_X_, and bending moments *M*_X_, *M*_Y_, used to calculate the resultant bending moment, *M*_T_. The lower limb of the 50th percentile MiL-Lx ATD was instrumented with a lower tibia load cell by the manufacturer to measure the same variables as above. The Revised Tibia Index (RTI) was calculated for both dummies using *F*_Z_ and *M*_T_ as described by Kuppa *et al*.^17^

**Figure 2:**
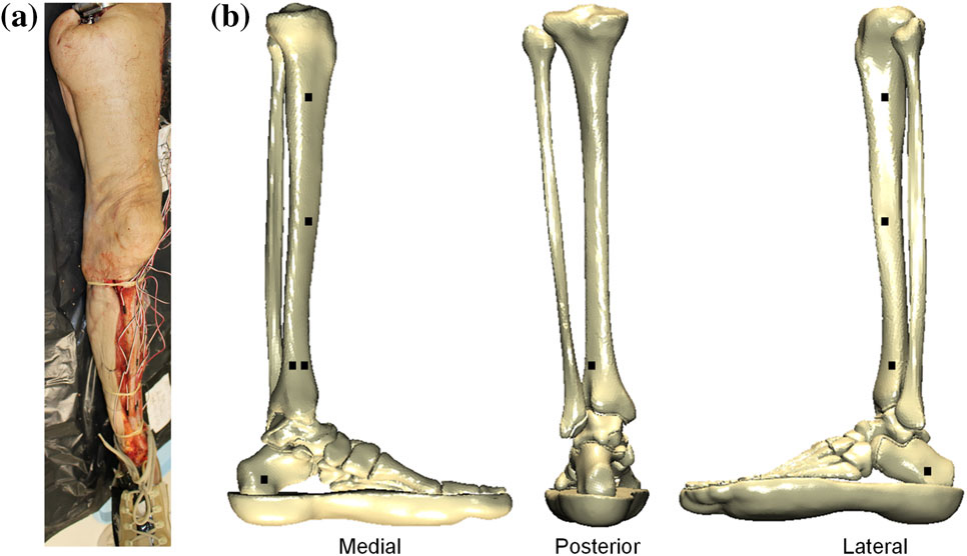
(a) An instrumented PMHS before being mounted on AnUBIS. (b) Schematic showing the location of strain gauges on the PMHS.

Ten male PMHS lower limbs of a mean (± SD) age of 53.4 ± 2.6 years (range 49–57 years), fresh frozen at − 20°, with no relevant known pathology that could affect the tissues of the lower limbs, were acquired from a licensed human tissue facility. The Tissue Management Committee of the Imperial College Tissue Bank ethics committee had granted ethical approval for this study (ethical approval number: 12-WA-0196). The limbs were scanned using computed tomography (CT) (Siemens Somatom Definition AS 64; Erlangen, Germany) before and after testing (pixel size of 0.5 × 0.5 mm at transverse slices of 1 mm) and were dissected and prepared.^21^ The anteromedial and anterolateral sides of the tibia were exposed and the side flaps were sutured posteriorly to allow for stain gauging. A wider area was isolated distally to allow for strain gauging of the posterior side of the bone as well as the anterior. Finally, the medial and lateral sides of the calcaneus were also exposed for strain gauging purposes. In total, ten strain gauges (model C2A-06-125LW-360, Vishay PG, Bradford, UK) were mounted using cyanoacrylate adhesive on the bones of the limbs at the anteromedial (× 1) and anterolateral (× 1) sides of the proximal tibia, at the anteromedial (× 1) and anterolateral (× 1) sides of the mid diaphysis of the tibia, at the anteromedial (× 2), anterolateral (× 1) and posteromedial (× 1) sides of the distal tibia, and at the medial (× 1) and lateral (× 1) sides of the calcaneus (Fig. 2). The strain gauges on the tibia were aligned to its long axis while the calcaneal strain gauges were perpendicular to the foot plate.

Fracture in the lower limbs was assessed using the CT scans performed after testing, the signals from the strain gauges, and the high-speed video footage. CT scans after testing were assessed to detect fractures that correspond to an AIS 2 + level of injury. Injury levels were quantified using the Abbreviated Injury Scale (AIS),^14^,^17^,^45^ the foot and ankle severity score (FASS),^18^ which is a better predictor of the clinical outcome of lower limb blast injuries,^37^ and the Sanders^39^ and Essex-Lopresti 11 classification for the calcaneal and the Orthopaedic Trauma Association classification (AO/OTA)^29^ for the tibial fractures.

## Results

The actual postures achieved in this study from all tests including both ATDs and PMHSs are shown in Table 1. The set-up of the ATDs could easily fit within the rig and was repeatable, and therefore standard deviations are not reported while the 90°–90° posture (Posture A) was adopted accurately by all surrogates. The difficulty of adopting the target angles with the PMHSs in some postures was because of the size restrictions of the rig which was originally developed to accommodate only the 90°–90° posture.

**Table 1:** Achieved mean (± 1 standard deviation) angles for postures 120°–120° and 120°–90° as defined in Fig. 1.

Surrogate limb	Posture	Knee angle	Ankle angle
H-III	120°–90°	115°	85°
	120°–120°	115°	115°
MiL-Lx	120°–90°	115°	85°
	120°–120°	115°	115°
PMHS	120°–90°	112° (± 3°)	82° (± 3°)
	120°–120°	113° (± 4°)	113° (± 4°)

**Figure 3:**
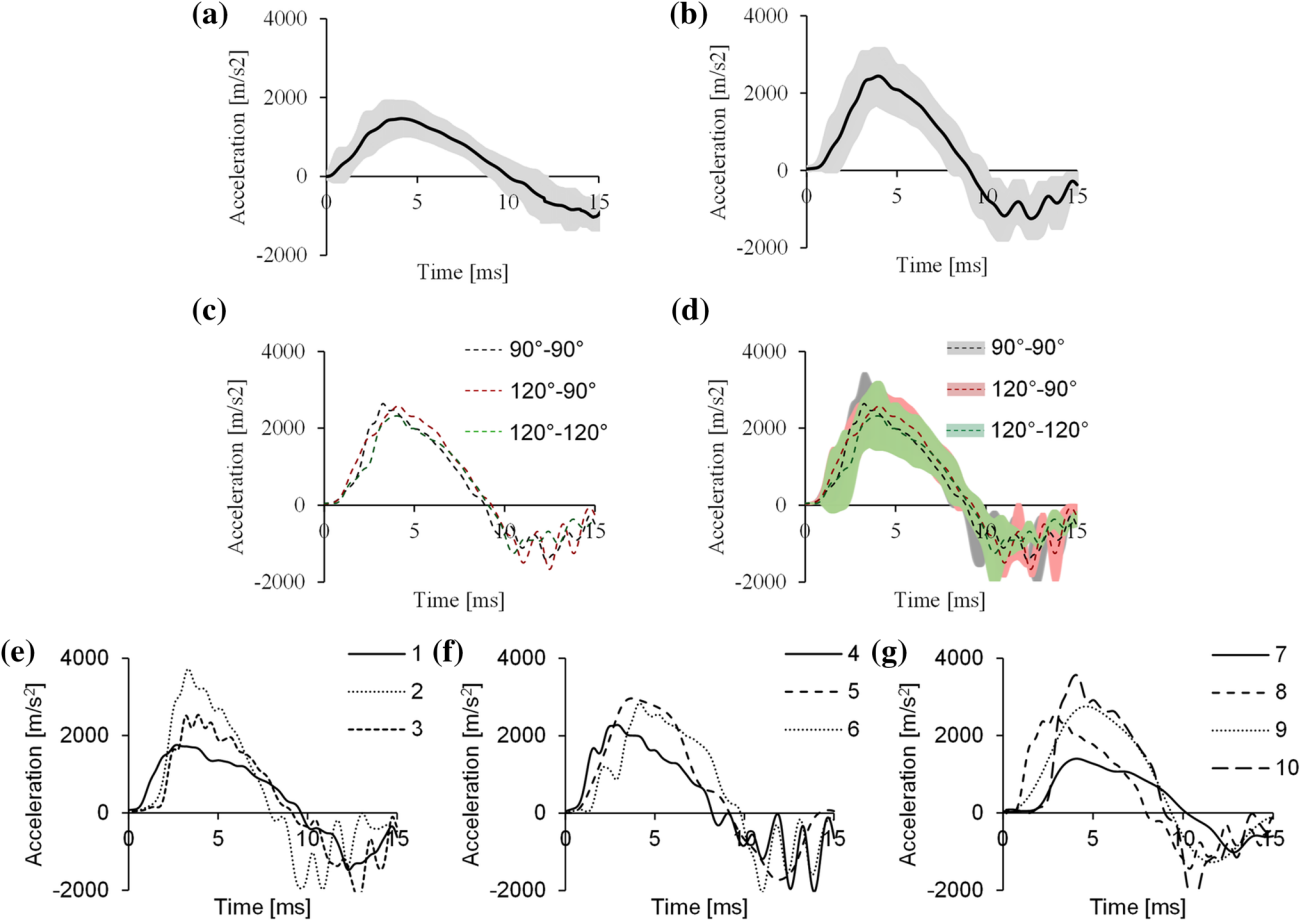
Mean acceleration pulses from (a) ATD and (b) PMHS tests. The shaded areas represent ± 1 standard deviation (SD). Comparison of mean acceleration pulses from PMHS tests per posture (c) without and (d) with ± 1SD (shaded area). Acceleration pulses from all PMHS tests set at posture (e) 90°–90°, (f) 120°–90°, and (g) 120°–120°.

The ATDs were exposed to a UBB loading of a mean (± SD) peak velocity of 8.7 (± 1.3) m/s reached at 10.8 (± 2.1) ms and a mean peak acceleration of 1802 (± 437) m/s^2^ (Fig. 3a). Of the ten PMHSs tested, three were set at posture 90°–90°, three at posture 120°–90°, and four at posture 120°–120°. They were exposed to a UBB loading of a mean (± SD) peak velocity of 12.7 (± 1.6) m/s reached within 8.8 (± 0.7) ms and a mean (± SD) peak acceleration of 2778 (± 608) m/s^2^ (Fig. 3b). The acceleration impulses per posture for the PMHS tests are shown in Figs. 3c and 3d and the data from these tests are summarised in Table 2.

**Table 2:** Summary of all PMHS tests and recorded data.

ID	Lower limb	Posture	Peak velocity (m/s)	Time to peak velocity (ms)	Peak *F*_H_ (kN)	Peak *F*_F_ (kN)
1	Right	90°–90°	9.9	9.6	8.3	2.7
2	Left	90°–90°	10.9	9.2	13.6	6.8
3	Right	90°–90°	13.7	9.8	13.75	9.2
4	Left	120°–90°	10.8	8.2	12.2	11.5
5	Left	120°–90°	14.4	9.2	8.7	8.7
6	Left	120°–90°	13.6	9.8	8.6	5.2
7	Left	120°–120°	8.6	10.2	8.7	4.1
8	Right	120°–120°	10.7	8.0	10.1	7.2
9	Left	120°–120°	14.0	9.3	11.6	7.1
10	Left	120°–120°	14.5	9.0	10.7	8.9

**Figure 4:**
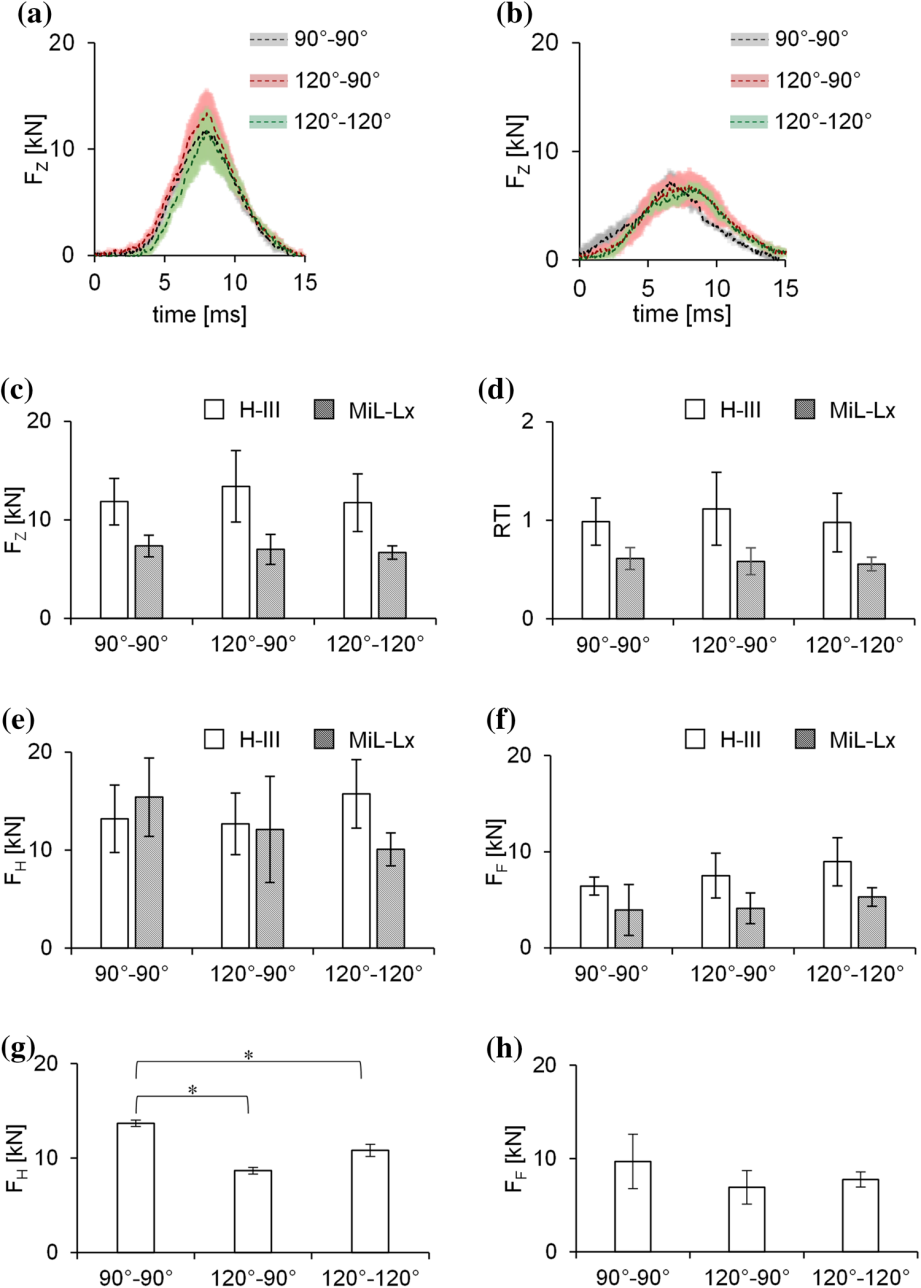
Average axial force, *F*_*Z*_, measured by the lower load cells of (a) the H-III and (b) MiL-Lx ATDs. The shaded areas represent ± 1 standard deviation (SD). Graphs show the mean peak values of (c) the measured *F*_*Z*_ and (d) calculated RTI from all tests and both ATDs. (e) Hind- and (f) fore-foot loading measured from the force sensors on the plate of AnUBIS (Fig. 1d). Comparison between peak (g) hind- and (h) fore-foot force values from injurious PMHS tests for each posture. Error bars represent 1 SD. Asterisk represents a statistical difference using ANOVA, analysed *post hoc* using a Games-Howell test with the significance level set at 0.05.

The axial force, *F*_Z_, measurements, recorded by the lower tibia load cell of the ATDs, are shown in Figs. 4a–4c. The peak values of the axial force, *F*_Z_, as well as the RTI, averaged for all postures are presented in Figs. 4d and 4e. There was no significant difference in any measured variable between postures (*p* > 0.05). The mean peak values of hind- and fore-foot force, *F*_H_ and *F*_F_, from PMHS tests in which a fracture was observed are presented in Figs. 4g and 4h.

**Figure 5:**
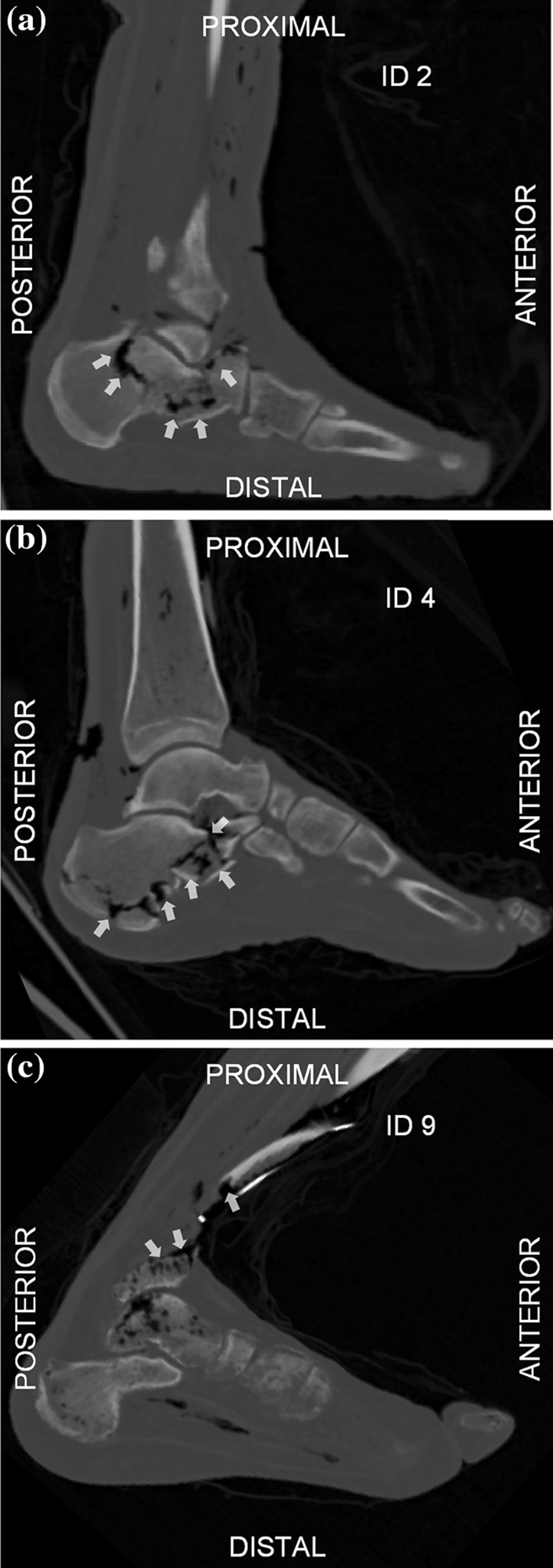
Sagittal CT scans showing fractures (white arrows) in PMHS lower limbs tested when set at posture (a) 90°–90°, (b) 120°–90°, and (c) 120°-120°.

Out of the ten tested PMHSs, seven sustained fractures of AIS 2 or higher (commonly noted as AIS2+); two set at posture 90°–90°, two at 120°–90°, and three at 120°–120°. The types and locations of fracture are presented in Table 3. Examples of radiographic evidence of the different types of fracture reported are shown in Fig. 5.

**Table 3:** Summary of all recorded fractures and their classification.

ID	Lower limb	Posture	AIS + 2	FASS	Fracture locations	Type of fracture
1	Right	90°–90°	No	–	–	–
2	Left	90°–90°	Yes	4	Calcaneus	Sanders IV
3	Right	90°–90°	Yes	4	Calcaneus	Sanders IV
4	Left	120°–90°	No	–	–	–
5	Left	120°–90°	Yes	4	Calcaneus	Tongue
6	Left	120°–90°	Yes	6	Distal tibia & Calcaneus	AO/OTA 43C3 Sanders I
7	Left	120°–120°	No	3	Calcaneus	Sanders I
8	Right	120°–120°	Yes	6	Distal tibia	AO/OTA 43C3
9	Left	120°–120°	Yes	6	Distal tibia	AO/OTA 43C3
10	Left	120°–120°	Yes	6	Distal tibia	AO/OTA 43C3

**Figure 6:**
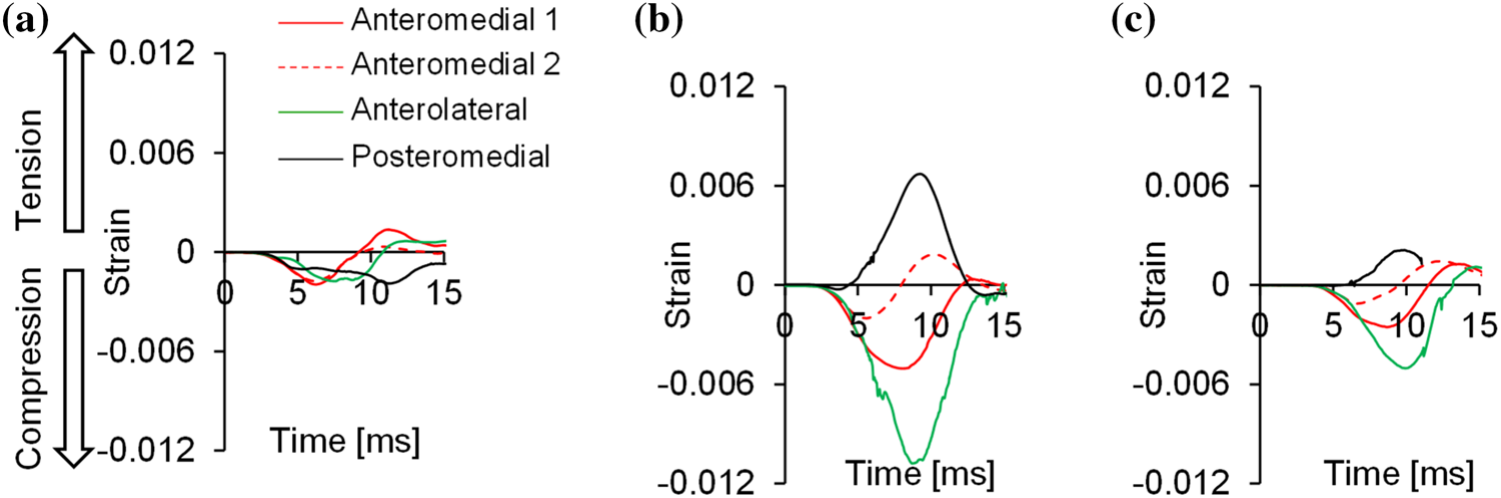
Distal tibia strains of the PMHS lower limbs that did not present an injury from testing. (a) Specimen 1 set at posture 90°–90°, (b) Specimen 4 set at posture 120°–90°, and (c) Specimen 7 set at posture 120°–120°.

Five Strain-gauge signals also revealed differences between postures. The shape of the distal tibial strain history was similar in the 120°–90° and 120°–120° postures but different to the 90°–90° posture (Fig. 6). While all sides of the distal tibia seem to be in compression at posture 90°–90°, its posteromedial side is consistently in tension in both postures 120°–90° and 120°–120° suggesting that a bending loading mode is dominating.

## Discussion

The effect of the seated posture of the lower limb in UBB was investigated in this study using both the H-III and MiL-Lx ATDs, and PMHSs. The surrogate limbs were exposed to UBB loading using a traumatic injury simulator. Load cell recordings of *F*_Z_ and *RTI* from testing the ATDs did not reveal statistical differences between postures suggesting that both the H-III and the MiL-Lx are not sensitive to posture change when the metrics mentioned above are selected for injury assessment. The PMHSs, however, sustained different fracture types and locations depending on posture, suggesting that posture indeed affects the respective mechanism of injury. In addition to the different types of fracture, posture 120°–120° resulted in more severe injuries than posture 90°–90°, as highlighted by the FASS values.

Using the results reported by Yoganandan *et al*.^44^ from matched-pair tests between the H-III ATD and PMHSs, the mean peak axial tibial forces from the lower load cell of the H-III ATD measured in this study correspond to a mean probability of an AIS2+ injury of 36, 51, and 35% for postures 90°–90°, 120°–90°, and 120°–120°, respectively. However, the axial tibial force measurements, as well as the *RTI* values, for both ATDs in this study were not found to be statistically different across postures. Therefore, despite the discrepancies above, the results from the ATDs suggest that all postures are of equal severity.

The biofidelity of the H-III at UBB loading has been assessed in the past with the ATD set at 90°–90° posture against data from PMHS tests performed on a pendulum, pneumatic piston, and a constant-velocity device.^4^ Similarly, during the development of the MiL-Lx, its response was compared to experiments conducted in a traumatic injury simulator and on PMHSs set at 90°–90°.^22^ As no such studies have been conducted at any other posture, their ability to predict the response of the lower limb in UBB when set at alternative seated postures is unknown. The results of the current study suggest that the ATDs cannot be used in conjunction with current IRFs to predict the probability of injury of the lower limb in alternative seated postures. Therefore, different metrics, posture specific IRFs, or posture-sensitive ATDs are required to assess UBB injury in alternative seated postures.

The ATDs were exposed to a different threat level than the PMHSs and therefore direct comparison under the same conditions was not possible. The H-III has been shown to overestimate the axial tibial force induced in UBB^4^,^5^,^17^,^22^,^26^ and therefore, to protect the H-III from damage, the target average peak velocity was 8.7 m/s at an average time to peak velocity of 10.8 ms, compared to an average velocity and time to peak velocity of 12.7 m/s and 8.8 ms in the PMHS tests. The MiL-Lx was exposed to the same threat as the H-III to allow for comparisons between them. This threat level produces no injury to the PMHSs and so in this study the PMHSs were tested at a higher threat level than that of the ATDs. As such, the effect of posture on injury was considered and discussed separately for ATDs and PMHSs.

The pulses of the PMHS tests had a wide SD as shown in Fig. 3. This was intentional to ensure that fractures would occur in all three postures with the number of specimens for this study (*n *= 10). Therefore, all cadavers were not exposed to statistically equivalent pulses, which is a limitation of this study. However, as shown in Table 2 and Fig. 3c, the mean pulses for each posture were similar (with maximum coefficient of variation less than 5% for both mean peak velocity and mean time to peak velocity values). Furthermore, based on the peak velocity and time to peak velocity characteristics shown in Table 2 and the pulses shown in Fig. 3, cadavers with IDs 2-4-8, and 3-5-6-9-10 were exposed to similar threats (with maximum coefficient of variation less than 7% for both peak velocity and time to peak velocity values in both groups). The fracture type discrepancies were mainly reported within these groups of cadavers suggesting that this behaviour is not related to the differences in the pulse characteristics used.

The use of a wedge was necessary to achieve the 120°–90° posture. In this setup, the hind-foot force sensor is still under the heel region while the forefoot force sensor is at an increased distance from the metatarsal heads of the surrogate limb compared to the other setups. The foot in the 120°–90° posture setup was oblique to the plate and the force measured from both force sensors was not perpendicular to the foot as in the other two setups. The force sensors, however, in all configurations recorded forces in the direction of the under-body blast loading, and that was considered as the most appropriate metric to compare between postures. In the rest of the postures, the feet of both ATDs and PMHSs were mounted so that the heel of the boot was centred above the hind-foot force sensor on the instrumented footplate. Since the fore-foot force sensor was mounted at a specific distance from the hind-foot force sensor, the fore-foot force sensor was best matching the metatarsal heads of the ATDs and the PMHSs fitted with a 10 UK size boot, but not the smaller sized PMHSs. This might explain the low fore-foot force measured when testing cadaver ID 1, which was the only one fitted with a 6 UK size boot. Therefore, the hind-foot force would be a more accurate metric to be used in case a 120°–120° posture-specific IRF is developed.

Testing the PMHSs in three different postures revealed differences in the measured plantar forces, the type and location of fractures, and the loading mode of the tibia. The peak hind-foot force from tests where an AIS 2 + injury was observed was significantly lower for postures 120°–120° and 120°–90° than for posture 90°–90° (Fig. 5). Furthermore, while posture 90°–90° led only to calcaneal fractures, one of the specimens set at posture 120°–90° and all injured specimens set at posture 120°–120° sustained distal tibia pilon fractures. According to the signal from the distal tibia strain gauges (Fig. 6), this can be attributed to the different loading experienced by the tibia; mainly compression when set at posture 90°–90° (negative signals), and both compression and bending (negative and positive signals) when set at postures 120°–90° and 120°–120°, respectively. The non-vertical orientation of the tibia to the ground appears to result in bending moments when loading is applied through the foot; this different mechanism of injury can explain the discrepancies shown in the fracture types between postures. A further analysis of the strain signals was not possible as it gets distorted during the injurious event with noise and artefacts been introduced by the excessive movement of bone fragments. That renders the identification of peak strain and time to peak strain challenging.

It is also likely that the limited amount of motion of the flexed ankle joint, when the lower limb is set at 120°–120°, determines the load pathway from the foot to the tibia. Furthermore, the geometry, the ankle joint stiffness and the loading of the surrounding ligaments are different when the foot is plantar-flexed at 120°. In such a setup, non-axial forces such as the friction between the boot and the force plate, might contribute to the moments experienced by the lower limb more than when the ankle angle is 90°. Previously, only computational studies investigated the hypotheses,^8^,^41^,^42^ with results contradicting the outcomes of this study. Dong *et al*.^8^ and Suresh *et al*.^41^ both predicted that a 120°–120° posture reduced the severity of UBB compared to 90°–90°. However, their prediction was based on the strain distribution of the bones of the foot and ankle when the strain at failure of tarsal bones at high loading rates is not known. Furthermore, the kinematic response of the computational models used was not validated against experimental data, and the initial position and orientation of the bones of the lower limb in various postures may not have been physiological. In addition, van der Horst *et al*.^42^ used a rigid body model and showed a reduction in tibial axial force when alternative seated postures, similar to 120°–90° and 120°–120°, were adopted. The reduction of the hind-foot force values when the knee joint extends from 90° to 90° to 120°–90° or 120°–120° was similar to the findings of this study. However, the probability of injury is not reduced as suggested by van der Horst *et al*.^42^; to the contrary, as bending moments become apparent, the mechanism of injury changes.

## Conclusion

This study showed that the posture of the lower limb, and more specifically the angles of the knee and ankle joints in the sagittal plane, affect the injury mechanism in UBB. Testing of PMHSs showed that the 120°–120° posture leads to fractures of higher severity that are more difficult to treat than the 90°–90° posture. Furthermore, the ATDs tested in this study were found to be insensitive to the changes in posture when RTI and Fz were used to assess injury and severity of injury in postures other than 90°–90° was found to be underestimated. These findings suggest that current ATDs and IRFs are not valid for assessing injury in postures 120°–90° and 120°–120°. Therefore, it is recommended that posture-specific IRFs are developed, and that care should be taken when interpreting and extrapolating results produced by ATDs when placed in postures other than 90°–90°, especially in vehicle qualification tests.
